# Primary Resistance to PD-1-Based Immunotherapy—A Study in 319 Patients with Stage IV Melanoma

**DOI:** 10.3390/cancers12041027

**Published:** 2020-04-22

**Authors:** Teresa Amaral, Olivia Seeber, Edgar Mersi, Stephanie Sanchez, Ioannis Thomas, Andreas Meiwes, Andrea Forschner, Ulrike Leiter, Thomas Eigentler, Ulrike Keim, Claus Garbe

**Affiliations:** 1 Center for Dermatooncology, Department of Dermatology, Eberhard Karls University of Tuebingen, 72076 Tuebingen, Germany; teresa.amaral@med.uni-tuebingen.de (T.A.);; 2 Portuguese Air Force—Health Care Direction, 1649-020 Lisbon, Portugal

**Keywords:** metastatic melanoma, primary resistance, checkpoint-inhibitors, combined immunotherapy, pseudoprogression

## Abstract

Background: Primary resistance to immunotherapy can be observed in approximately 40–65% of the stage IV melanoma patients treated with immune checkpoint inhibitors. A minority of the patients receive a second-line therapy, and the clinical benefit is small. Patients and methods: Stage IV melanoma patients treated with first-line PD-1-based immunotherapy between January 2015 and December 2018 were investigated. Primary resistance was defined as progressive disease (PD) at the time of the first tumor assessment after starting immunotherapy. Patients with complete response, partial response, and stable disease were classified as having disease control (DC). Overall survival (OS) and progression-free survival (PFS) were evaluated by Kaplan–Meier estimator. Univariate and multivariate logistic regression analyses were performed to determine prognostic factors associated with OS. Results: Three hundred and nineteen patients were included, and 40% had primary resistance to immunotherapy. The median follow-up time was 22 months. Patients with primary resistance had 1-, 2-, and 3-year OS rates of 41%, 15%, and 10%, respectively, compared to 91%, 81%, and 65% for the patients who achieved DC. The following independently significant prognostic factors for OS were identified: protein S100B level and primary tumor localization. There was a statistically significant difference for OS (*p* < 0.0001) but not for PFS (*p* = 0.230) when analyzing risk groups formed with a combination of these two variables (low-, intermediate-, and high-risk subgroups). Conclusions: Melanoma patients with primary resistance to immunotherapy have a dismal prognosis. Response at the first tumor assessment after starting immunotherapy is a stronger prognostic factor for the further course of the disease than pretreatment risk factors.

## 1. Introduction

Immunotherapy with checkpoint inhibitors is currently the most effective therapy for metastatic melanoma, achieving high remission rates and long-term survival [1]. These therapies include ipilimumab, a cytotoxic T-lymphocyte-associated protein 4 (CTLA-4) antibody, nivolumab and pembrolizumab, both programmed cell death protein 1 (PD-1) antibodies, and the dual combination of anti-PD-1 and anti-CTLA-4 antibodies nivolumab plus ipilimumab. Results from studies investigating these therapies in melanoma patients are available [2,3,4]. Long-term survival data have also been published, specifically the 5- and 10-year overall survival (OS) rates for ipilimumab monotherapy, and 5-year OS rates for nivolumab, and the combination of nivolumab with ipilimumab [4,5,6]. The latest update showed that the 5-year OS survival rate for nivolumab and ipilimumab was 52%, for nivolumab monotherapy was 44%, and for ipilimumab monotherapy was 26% [4].

Despite improvement in survival outcomes compared with the past (e.g., chemotherapy), primary resistance to checkpoint inhibitor therapy exists, and a considerable number of patients still do not derive benefit from these therapies [7,8]. Primary resistance is typically assumed in the clinical practice if tumor progression is observed at the first tumor assessment after therapy starts, which in our center takes place around week 12 (+/−5 days). It is observed in a rather high percentage of patients, estimated to be between 40% and 65%, depending on whether patients receive first-line immunotherapy or immunotherapy after progression under other systemic therapies [3,9,10]. Higher percentages were observed when patients were treated with ipilimumab monotherapy [2,11].

Resistance to immunotherapy can be classified as primary (or innate) and secondary or acquired [12,13,14,15]. Some authors also refer to an intermediate phenotype that is adaptive resistance [16]. Clinically, resistance mechanisms to immunotherapy can be grouped into those that always preclude response to immunotherapy and those that appear later, allowing tumor escape and progression after an initial benefit. However, the molecular mechanisms of resistance to immunotherapy involving the host, the tumor, and the tumor microenvironment can overlap and be present at different timepoints of the course of the disease.

Regarding primary resistance to immunotherapy, which is the focus of our analysis, the following resistance mechanisms have been described: (a) diminished sensitivity to the INF- signaling pathway [8,16,17]; (b) insufficient T-cell activation or absence of T-cells in the tumor microenvironment [18,19,20]; (c) increased infiltration of T-regulatory cells [21,22,23]; (d) upregulation of immunosuppressive markers [24,25]; (e) insufficient antigen presentation and/or antigen recognition, due to, for example, low tumor mutation burden, loss of MHC class I and β-2 microglobulin, or absence of neo-antigen presentation [17,26,27,28,29].

In the present study, we focus on the clinical outcomes of stage IV melanoma patients with primary resistance to first-line PD-1-based immunotherapy, specifically pembrolizumab, nivolumab, and nivolumab plus ipilimumab. We evaluate the course of the disease in patients prospectively registered in the Central Malignant Melanoma Registry (CMMR) of the German Dermatological Society, and treated between January 2015 and December 2018 at the University Hospital Tübingen. We addressed the following questions: (1) Which factors are associated with the development of primary resistance? (2) How does survival of patients with primary resistance compare to those with disease control (complete response (CR), partial response (PR), and stable disease (SD))? (3) Did the patients with primary resistance to PD-1-based immunotherapy receive further therapies, and if so, which therapies were offered and what was the outcome?

## 2. Patients and Methods

### 2.1. Patients Cohort

Three hundred and nineteen patients with stage IV melanoma treated with first-line anti-PD-1 antibodies immunotherapy were included. These patients had available data on the type of response at the time of the first tumor assessment after starting immunotherapy, and also data that allowed us to identify the best overall response to immunotherapy. The Ethics Committee of the Medical Faculty of the University of Tübingen approved this study (approval number 676/2016BO2).

All patients included signed the patients’ informed consent and were prospectively recorded by the German Central Malignant Melanoma Registry (CMMR). Clinical data were obtained from the clinical records from the University Hospital Tübingen, documented in an open source database, Epi Info™, and later merged into a final SPSS^®^ file. The following variables were recorded: gender, date of birth, date of stage IV diagnosis, stage at initial diagnosis according to the American Joint Committee on Cancer (AJCC) version 8 [30], localization and histopathological characteristics of the primary tumor, *BRAF* mutation status, protein S100B level, lactate dehydrogenase (LDH) level at the time of stage IV diagnosis, localization and number of metastatic organs, type of systemic therapy for stage IV disease and respective start and end dates, response at the first tumor assessment after systemic therapy start, best overall response to systemic therapy, and time of last follow-up or death from any cause.

Primary resistance was defined as progressive disease (PD) at the time of first tumor assessment after immunotherapy start. In our center, this is performed after 12 weeks (+/−5 days). This evaluation was performed using RECIST 1.1 [31]. Patients with CR, PR, and SD were considered to have disease control (DC). Best overall response to first-line immunotherapy was defined as the best response—intracranial and extracranial—that patients achieved during the time they were treated. Taking that into consideration, patients for whom the best overall response was PD were, by definition, patients with primary resistance. These patients did not continue to receive immunotherapy, since the clinical evaluation also determined that they were not deriving benefit from the ongoing therapy.

Pseudoprogression was considered for patients who were classified as having PD by RECIST 1.1 [31] at the time of first assessment after immunotherapy start but, due to clinical benefit, continued receiving immunotherapy and had a response later in the course of their disease. These patients were not considered as primary resistant and were included in the group of disease control.

### 2.2. Statistical Analysis

Statistical analysis was performed using the statistical program for social sciences SPSS Version 25 (IBM, New York, NY, USA). STATA^®^ v15 (StataCorp LLC, College Station, TX, USA) was used to generate the final version of the Kaplan–Meier survival curves.

Descriptive statistical analyses, frequency tables, and chi-square tables were used to characterize the patients’ population. Variables with missing information were excluded from the respective analysis. Follow-up time was defined as the time between the date of stage IV diagnosis and the date of the last follow-up or death from any cause. Survival analyses were performed according to the Kaplan–Meier method. In addition, the 1-, 2-, and 3-year survival rates were calculated with a 95% confidence interval. Factors that were significant in the univariate analysis were included into the multivariate logistic regression analysis. The level of significance was 0.05 (two-sided) in all analyses. The cut-off date for data analysis was March 2019.

## 3. Results

### 3.1. Univariate and Multivariate Analysis

Table 1 shows characteristics of the study population: 192 patients (60%) had disease control (SD, PR, CR) and 127 (40%) patients had primary resistance. The median age of the patients at the time of stage IV melanoma diagnosis was 68 years; interquartile range (IQR) (56–77). Age was not associated with primary resistance. Thirty-five patients (11%) had more than 3 organs with metastases at the time of immunotherapy beginning and 292 patients (89%) had 1–3 organs with metastases. Sixty-three (19%) patients had brain metastases and 118 (36%) patients had liver metastases. The number of organs involved, the presence of brain metastases, and the presence of liver metastases were not associated with primary resistance in our analysis.

In our cohort we had slightly more men (60%) than women (40%). In the univariate analysis, sex was a statistically significant factor associated with primary resistance, with male patients having better outcomes than female patients. In the multivariate logistic regression analysis, sex was not a statistically significant factor.

Tumor localization was significantly associated with primary resistance. Tumors of the extremities, including acral melanomas, showed significantly increased primary resistance. Tumor localization remained a significant factor in multivariate logistic regression analysis. The histological subtype was also associated with primary resistance. Primary resistance was found especially in acral lentiginous melanoma, mucosal melanoma, and other melanomas. In the multivariate logistic regression analysis, however, the histological subtype was not a significant factor.

Another significant factor in the univariate analysis was an elevated level of the tumor marker protein S-100B, which was associated with a significantly increased primary resistance, both in the univariate and in the multivariate logistic regression analysis. An elevated LDH level was also significantly associated with increased primary resistance in the univariate analysis, but was not significant in the multivariate logistic regression analysis. The following variables were not significant either in univariate analysis or in multivariate analysis: stage at initial diagnosis, number of metastatic organs, presence of brain metastases, presence of liver metastases, and *BRAF* mutation status.

Table S1 shows characteristics of the study population where the primary resistance group includes patients with PD at the first evaluation after starting immunotherapy and patients with SD with a duration of less than 6 months (*n* = 169), and the DC group includes patients with CR, PR, and SD with a duration of more than 6 months (*n* = 190). The results are similar to the ones described above, except that the number of organs with metastases is a statistically significant factor in the univariate analysis and LDH level is no longer statistically significant.

### 3.2. Survival Analysis

The median overall survival (OS) and the 1-year, 2-year, and 3-year OS rates are summarized according to the best response in Table 2. The 2-year OS rates were 96% for patients with CR, 84% for patients with PR, and 64% for patients with SD. Patients with primary PD had a 2-year OS rate of 15%. The corresponding progression-free survival (PFS) rates are summarized in Table 3. Here, 2-year PFS was 81% for CR, 63% for PR, 22% for SD, and 3% for PD. The 2-year OS rate in the disease control (SD + PR + CR) group was 81% versus 15% in patients with primary resistance. As for the 2-year PFS rate it was 56% versus 3%, respectively.

**Figure 1:**
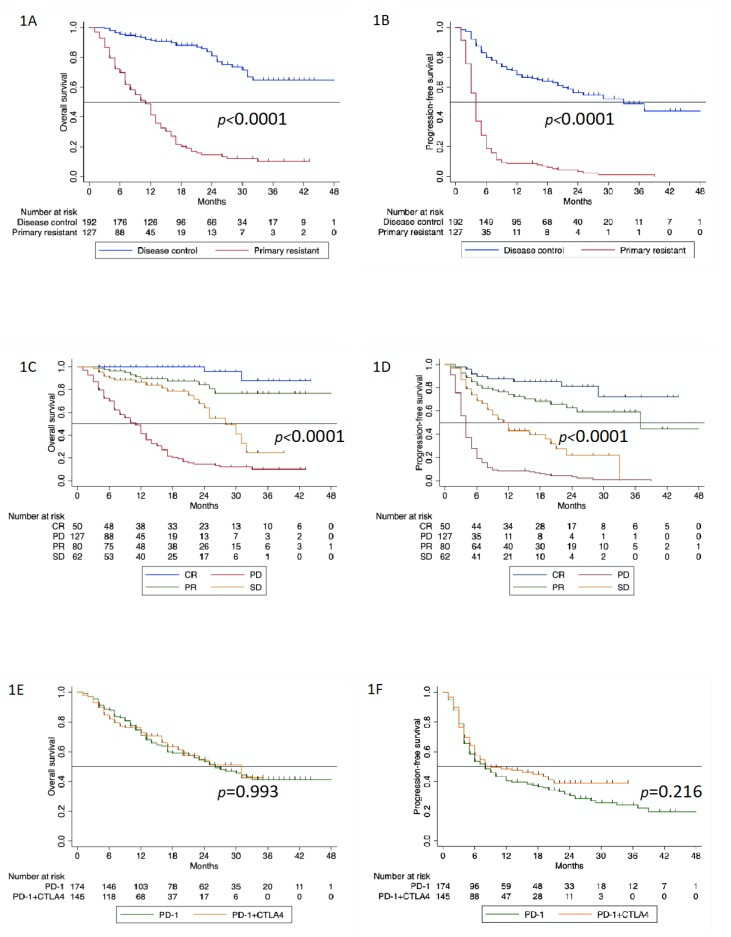
(**A**). Overall survival according to response to first-line PD-1-based immunotherapy (*p* < 0.0001); (**B**). progression-free survival according to response to first-line PD-1-based immunotherapy (*p* < 0.0001); (**C**). overall survival according to best overall response to first-line PD-1-based immunotherapy (*p* < 0.0001); (**D**). progression-free survival according to best overall response to first-line PD-1-based immunotherapy (*p* < 0.0001); (**E**). overall survival according to the type of first-line PD-1-based immunotherapy (*p* = 0.993); (**F**). progression-free survival according to the type of first-line PD-1-based immunotherapy (*p* = 0.216).

A statistically significant difference can be seen in OS when patients are classified as having primary resistance or disease control at the time of first tumor assessment after starting immunotherapy (Figure 1A; *p* < 0.0001). The same is true for PFS (Figure 1B; *p* < 0.0001). After three years, a plateau was formed for the group with disease control at a level of 65%, while the PFS rate for primary resistance decreased to 10%. After three years, a certain plateau formation around a 45% PFS rate was also visible in patients with disease control, while in patients with primary resistance, the PFS rate dropped to 1%.

The OS curves according to Kaplan and Meier show that in cases of CR and PR, the survival remained largely stable after two years. This was not the case in patients that achieved SD, where there was a relatively steep drop in the survival curve after 18 months, leading to OS rates very close to those in patients with PD (Figure 1C; *p* < 0.0001). In the PFS analysis, there were even clearer differences between CR and PR. After the first year, there was a clear drop in PFS rates for PR compared to CR. After three years, patients with SD had approximately the same survival rates as patients with PD (Figure 1D; *p* < 0.0001). 

Table S2 shows the patients characteristics for the whole cohort, considering the type of first-line immunotherapy received. One hundred and seventy-four patients received monotherapy with anti-PD-1 (nivolumab or pembrolizumab), while 145 patients were treated with the combination of nivolumab plus ipilimumab. The survival curves for OS overlapped completely, and in our cohort, there was no apparent benefit for the combination treatment (Figure 1E; *p* = 0.993). The survival curves for PFS separated approximately eight months after the start of treatment with a slightly more favorable course for the combined regimen, but this difference was not statistically significant (Figure 1F; *p* = 0.216). 

In order to evaluate whether primary resistance can be predicted based on pre-existing risk factors, three subgroups were defined, considering the two factors that were significant in the multivariate regression analysis (i.e., primary tumor localization and protein S-100B level) (Figure S1). The subgroups were defined as follows: no risk factor (low-risk), one risk factor (intermediate-risk) and two risk factors (high-risk). The survival analysis showed that OS overlapped for the low and intermediate subgroups, while a significantly less favorable survival was observed for high-risk patients (Figure S1A; *p* < 0.0001). There was no significant difference in terms of PFS (Figure S1B; *p* = 0.230).

Finally, the analysis where the primary resistance group included patients with PD and SD for less than six months and the DC group included patients with CR, PR, and SD for more than six months (Figure S2A,B) showed that the difference in terms of OS and PFS remained statistically significant (*p* < 0.0001).

### 3.3. Second-Line Therapies and Outcomes

Tables S3 and S4 show the type of second-line therapy in patients with primary resistance, and also the best overall response achieved, according to the *BRAF* mutation status. Approximately 50% (*n* = 63) of the patients with primary resistance to immunotherapy received a second-line therapy. Sixty-four patients did not receive further systemic therapies. Twenty-one patients had tumors harboring a *BRAFV600E/K* mutation, 20 patients had *BRAF* wild-type tumors, and in 22 patients, there was no information regarding *BRAF* mutation status. The majority of the patients with *BRAFV600E/K* mutation (17/21) received targeted therapy with *BRAF* plus MEK inhibitors, three received immunotherapy, and one patient chemotherapy. Patients with *BRAF* wild-type tumors received in equal number immunotherapy (10/20) and chemotherapy (10/20).

Information on the best overall response for the second-line systemic therapy was available for 58 patients. In five patients, this information was not available. Patients with tumors harboring a *BRAFV600E/K* mutation received predominantly targeted therapy which resulted in a high response rate (CR or PR) of 63%. In patients with *BRAF* wild-type tumors treated either with second-line immunotherapy or chemotherapy, the response rate was only 11% (Table S3). 

### 3.4. Pseudoprogression

In our cohort (*n* = 319), we identified six patients with pseudoprogression. Of these six patients, five showed initial PD but later achieved SD as best overall response to immunotherapy, and in one patient after initial PD, the best overall response to immunotherapy was CR. 

## 4. Discussion

Our study shows that patients with primary resistance and tumor progression at the time of first tumor assessment after starting immunotherapy have a highly significantly unfavorable survival rate as compared to those who achieve disease control. Response at the time of first tumor assessment after starting immunotherapy is a better predictive factor for survival than other pretreatment risk factors for the development of primary resistance. Achieving an objective remission (CR or PR) is decisive for favorable OS. The median OS for patients with SD is significantly better than for patients with primary resistance (28 months versus 11 months). After three years, however, the survival curves converge strongly at an unfavorable level. This convergence is even more pronounced for PFS. 

In the multivariate logistic regression analysis, only two significant risk factors for primary resistance to immunotherapy were identified. These were primary tumor localization and an elevated level of protein S-100B. In the univariate analysis, an elevated level of LDH was also a significant factor, but this did not remain significant in the multivariate analysis. The decisive factor here may be that the LDH value and the protein S-100B value usually increase in parallel, and that the S-100B value increases earlier and in more patients. 

In the univariate analysis, the histological subtype was also a significant factor. Here there is an overlap with tumor localization, since ALM is more commonly seen in the extremities, and mucosal melanomas were classified in the other localizations group. The higher discriminatory power was observed for the tumor localization. There is also a relationship between tumor localization and sex, as melanomas in the extremities occur more frequently in females, and females have a less favorable response to immunotherapy than males [32,33,34]. Accordingly, sex was a significant risk factor in the univariate analysis but not in the multivariate analysis. 

The definition of risk groups considering the two risk factors that remained significant in the multivariate analysis (i.e., primary tumor localization and protein S-100B) showed a relatively low predictive value. We observed a statistically significant difference in terms of OS between high- and intermediate- and low-risk groups, whereas this difference was not observed in PFS. 

The three-year PFS and three-year OS rates reported here for PD-1 monotherapy and the combination of nivolumab plus ipilimumab are lower than those reported in the CheckMate 067 trial [35]. This might be partially explained by the selection of the patients included in that study compared to the unselected population in our cohort. As an example, patients with (active) brain metastases are typically excluded from clinical trials. In fact, only 3.6% of the patients included in the CheckMate 067 trial had brain metastases compared to 19% in our study. In our cohort, 36% of the patients also had liver metastases, which is associated with worse response to immunotherapy [36,37]. The percentage of patients that had elevated LDH is similar in both reports (32% in our cohort vs. 36% in the CheckMate 067 trial). In the CheckMate 067 trial, the S100B levels, which are a known prognostic factor [38,39], were not reported; in our study, 44% of the patients had elevated S100B. Together, these aspects define a collective of patients that probably had a worse prognosis compared to the patients included in the clinical trial.

In our cohort, there was no difference in terms of OS between patients treated with PD-1 monotherapy and those receiving nivolumab plus ipilimumab. This might be related to the median follow-up time of only 22 months, shorter than the last update from the CheckMate 067 trial, where the difference between combined immunotherapy and monotherapy was clearer with a five-year follow-up [4]. In our cohort, a significantly higher proportion of patients with *BRAFV600E/K* mutation received combined immunotherapy (*p* = 0.003). This subgroup seemed to respond better to combined immunotherapy compared to PD-1 monotherapy [4,40], and this might explain why we started to see a separation of the PFS curves. Possibly with a longer follow-up, a difference in OS can be expected. 

The absence of difference in terms of OS in these two subgroups is probably also linked to the fact that they were not homogenous, with a selection bias regarding the type of immunotherapy. Older patients, who seem to respond better to immunotherapy [41], received predominantly PD-1 monotherapy (*p* = 0.007), patients with more than three metastatic organs received preferably combined immunotherapy (*p* = 0.043), and a significantly higher proportion of patients with brain metastasis were also treated with nivolumab plus ipilimumab (*p* = 0.018). 

Only 50% of the patients with primary resistance to immunotherapy received a second systemic therapy, similar to the percentage of patients reported in other series receiving a second-line therapy [42]. In our cohort, patients with *BRAFV600E/K* mutation received predominantly targeted therapy, and in this subgroup, 63% of patients had a response (CR or PR). This was slightly higher than previously published [43], but in our cohort, only 21 patients with *BRAFV600E/K* mutation received second-line therapy, and therefore the outcomes need to be interpreted cautiously. Nevertheless, our group and others have already demonstrated that patients with *BRAFV600E/K* first-line immunotherapy followed by targeted therapy, similar to what patients in this cohort received, seem to have better outcomes than the inverse sequence [44,45]. The high response rate in our cohort might be explained by this favorable therapy sequencing.

On the other hand, for patients with *BRAF* wild-type tumors, only one patient responded to second-line therapy. Again, the number of patients was low (*n* = 20), but these results show that a second-line therapy in the *BRAF* wild-type cohort is not possible in a high number of patients and, when possible, still has a small impact on survival. 

Strengths of this investigation are the fact that the data included was from a German certified skin cancer center with high standards for data quality. Three hundred and nineteen patients were analyzed, which is a large cohort of patients with stage IV melanoma managed with PD-1-based immunotherapy in a routine clinical setting. This high number of patients allowed us to perform subgroup analyses, with results of reasonable sensitivity. Further, this study provides follow-up data covering a period of up to 22 months.

The study limitations are related to its retrospective and monocentric design. Patients included were those receiving first-line immunotherapy for stage IV melanoma and for whom a response to therapy was documented. Since no other selection criteria were applied, the heterogeneity of the study population might have contributed to the differences observed in survival. Another limitation is the absence of histological confirmation of progressive disease in all patients with primary resistance. This approach is currently changing and, in the future, it would certainly be of value to include other factors in the definition of primary resistance.

## 5. Conclusions

Patients with progressive disease at the first tumor assessment after starting first-line PD-1-based immunotherapy have a very unfavorable prognosis. Predicting primary resistance based on pre-existing risk characteristics is possible only to a limited extent. Response at time of first tumor assessment after starting immunotherapy is a stronger predictive factor. In future analysis, other factors, namely histological and molecular characterization of the progressive lesions, should be included in the definition of primary resistance.

## Figures and Tables

**Table 1:** Patients characteristics, univariate and multivariate analysis for the whole cohort, according to best overall response to first-line PD-1-based immunotherapy.

Characteristics	ICI Cohort *n* = 319 *n* (%)	*n* (%)		Univariate Analysis $\chi^{2}$ Test 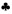	Multivariate Logistic Regression Analysis
		Primary Resistance *n* = 127 (40)	DC (CR, PR, SD) *n* = 192 (60)		
**Age Distribution**				0.732	
Median (years [IQR])	68 (56–77)	65 (55–78)	68 (56–77)		
<60y	101 (32)	37 (29)	64 (33)		
60y–75y	114 (36)	47 (37)	67 (35)		
>75y	104 (32)	43 (34)	61 (32)		
**Sex**				**0.049**	0.822
Male	192 (60)	68 (54)	124 (65)		
Female	127 (40)	59 (46)	68 (35)		
Tumor localization *				**0.000**	**0.001**
Head and neck	54 (22)	12 (13)	42 (27)		
Trunk	73 (29)	18 (20)	55 (34)		
Extremity	109 (43)	54 (59)	55 (34)		
Other	15 (6)	7 (8)	8 (5)		
**Histological subtype** *				**0.007**	0.452
SSM	76 (32)	31(37)	45 (29)		
NM	72 (30)	18(21)	54 (35)		
LMM	13 (6)	0	13 (9)		
ALM	30 (12)	15 (18)	15 (10)		
Mucosal	15 (6)	7 (8)	8 (5)		
Other	32 (14)	14 (16)	18 (12)		
**Stage at initial diagnosis** *				0.114	
I	48 (17)	19 (18)	29 (17)		
II	84 (31)	25 (23)	59 (35)		
III	95 (35)	38 (37)	57 (34)		
IV	47 (17)	24 (22)	23 (14)		
**Number of organs with metastases**				0.098	0.470
1-3	285 (89)	109 (86)	176 (92)		
>3	34 (11)	18 (14)	16 (8)		
**Brain metastases**				0.618	
No	258 (81)	101 (79)	157 (82)		
Yes	61 (19)	26 (21)	35 (18)		
**Liver metastases**				0.139	
No	204 (64)	75 (59)	129 (67)		
Yes	115 (36)	52 (41)	63 (33)		
***BRAF* mutation** *				0.844	
*BRAF*mut	88 (45)	32 (44)	56 (46)		
*BRAF*wt	106 (56)	40 (56)	66 (54)		
**LDH level** *				**0.029**	0.532
Normal	190 (68)	67 (60)	123 (73)		
Elevated	90 (32)	44 (40)	46 (27)		
**S100B level** *				**0.000**	**0.008**
Normal	157 (56)	44 (40)	113 (65)		
Elevated	125 (44)	65 (60)	60 (35)		

* patients with no information available were excluded in the respective analysis; IQR = interquartile range; 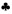 Chi-square test performed between the two groups—primary resistance and disease control; ICI = immune-checkpoint inhibitors cohort—145 patients received first-line treatment with nivolumab plus ipilimumab and 174 received antiPD-1 antibodies monotherapy (nivolumab *n* = 46 and pembrolizumab *n* = 128).; y = years; SSM = superficial spreading melanoma; NM = nodular melanoma; LMM = lentigo malignant melanoma; ALM = acral lentiginous melanoma; *BRAF*mut = presence of *BRAFV600E/K* mutation; *BRAF*wt = *BRAF* wild-type; LDH = lactate dehydrogenase; S100B = tumor marker protein S100B. *p*-values that are statistically significant are noted in bold.

**Table 2:** Median overall survival and overall survival rates for patients receiving first-line PD-1-based immunotherapy according to best overall response and type of immunotherapy.

Best Response	Median OS (Months; 95% CI)	OS (%; 95% CI)		
		1-Year	2-Year	3-Year
CR *n* = 50 (15.7%)	not reached	100%	95.7 (87.3–100)	87.7 (70.8–100)
PR *n* = 80 (25.1%)	not reached	89.5 (82.1–96.9)	84.4 (74.4–94.4)	84.4 (74.4–94.4)
SD *n* = 62 (19.4%)	28 (22.9–33.1)	86.3 (77.5–95.1)	63.8 (47.7–79.9)	24.6 (2.6–46.5)
PD *n* = 127 (39.8%)	11 (9.0–13.0)	41.3 (31.9–50.7)	14.7 (7.4–22.0)	10.1 (3.4–16.8)
DC *n* = 192 (60.2%)	not reached	91.3 (87.0–95.6)	81.0 (73.7–88.3)	64.6 (53.2–76)
PD-1 monotherapy *n* = 174 (66.2%)	26 (19.7–32.3)	71.1 (64.0–78.2)	53.3 (45.1–61.5)	41.3 (32.1–50.5)
PD-1 + CTLA4 *n* = 145 (54.6%)	31 (17.2–44.8)	72.8 (65–80.6)	54.5 (42.9–66.1)	42.5 (24.1–60.9)

OS = overall survival; CR = complete response; PR = partial response; SD = stable disease; PD = progressive disease; DC = disease control (CR + PR + SD); PD-1 monotherapy = nivolumab or pembrolizumab; PD-1 + CTLA4 = nivolumab plus ipilimumab.

**Table 3:** Median progression-free survival and progression-free survival rates for patients receiving first-line PD-1-based immunotherapy according to best overall response and type of immunotherapy.

Best Response	Median PFS (Months; 95% CI)	PFS (%; 95% CI)		
		1-Year	2-Year	3-Year
CR *n* = 50 (15.7%)	Not reached	87.6 (78.4–96.8)	81.2 (68.9–93.5)	72.2 (52.2–92.2)
PR *n* = 80 (25.1%)	37 (14.97–59.03)	74.4 (64.2–85.0)	62.7 (50.0–75.4)	62.7 (50.0–75.4)
SD *n* = 62 (19.4%)	12 (8.97–15.03)	43.0 (29.3–56.7)	21.8 (6.3–37.3)	-
PD *n* = 127 (39.8%)	4 (3.56–4.44)	8.7 (3.8–13.6)	3.2 (0–6.5)	1.1 (0–3.1)
DC *n* = 192(60.2%)	33 (20.4–45.6)	68.1 (61.0–75.2)	56.2 (51.8–64.8)	48.7 (37.7–59.7)
PD-1 monotherapy *n* = 174 (66.2%)	8 (5.5–10.5)	40.3 (32.7–47.9)	30.5 (23.1–37.9)	24.1 (16.3–31.9)
PD-1 + CTLA4 *n* = 145 (54.6%)	9 (1.8–16.2)	48.5 (40.1–56.9)	39 (29.2–78.8)	-

PFS = progression-free survival; CR = complete response; PR = partial response; SD = stable disease; PD = progressive disease; DC = disease control (CR + PR + SD); PD-1 monotherapy = nivolumab or pembrolizumab; PD-1 + CTLA4 = nivolumab plus ipilimumab.

## Supplementary Materials

The following are available online at https://www.mdpi.com/2072-6694/12/4/1027/s1, Figure S1: (**A**) Overall survival according to risk groups (low, intermediate, and high) (*p* < 0.0001). The factors used were primary tumor localization and protein S-100B level. The subgroups were defined as follows: no risk factor (low), one risk factor (intermediate), and two risk factors (high). (**B**) Progression-free survival according to risk groups (low, intermediate, and high) (*p* = 0.230). The factors used were primary tumor localization and protein S-100B level. The subgroups were defined as follows: no risk factor (low), one risk factor (intermediate), and two risk factors (high); Figure S2: (**A**) Overall survival for the disease control group (complete response, partial response, and stable disease for more than 6 months) and primary resistance (progressive disease and stable disease for less than 6 months). (**B**) Progression-free survival for the disease control group (complete response, partial response, and stable disease for more than 6 months) and primary resistance (progressive disease and stable disease for less than 6 months); Table S1: Patients characteristics and univariate analysis for the whole cohort. In this analysis, the primary resistance group includes progressive disease at the time of first tumor response evaluation after immunotherapy plus stable disease for less than 6 months. The disease control group includes complete response, partial response, and stable disease for longer than 6 months; Table S2: Patients characteristics and univariate analysis for the whole cohort according to type of first-line immunotherapy; Table S3: Second-line therapies in patients with primary resistance considering *BRAF* mutation status; Table S4: Best overall response to second-line therapies in patients with primary resistance considering *BRAF* mutation status.
