# Supplementary material for: Primary Resistance to PD-1-Based Immunotherapy—A Study in 319 Patients with Stage IV Melanoma
Source: Cancers (Basel). 2020 Apr 22;12(4):1027. doi: 10.3390/cancers12041027 (PMC7226601; doi:10.3390/cancers12041027)
Supplement: Supplementary file 1 [file cancers-12-01027-s001.pdf]

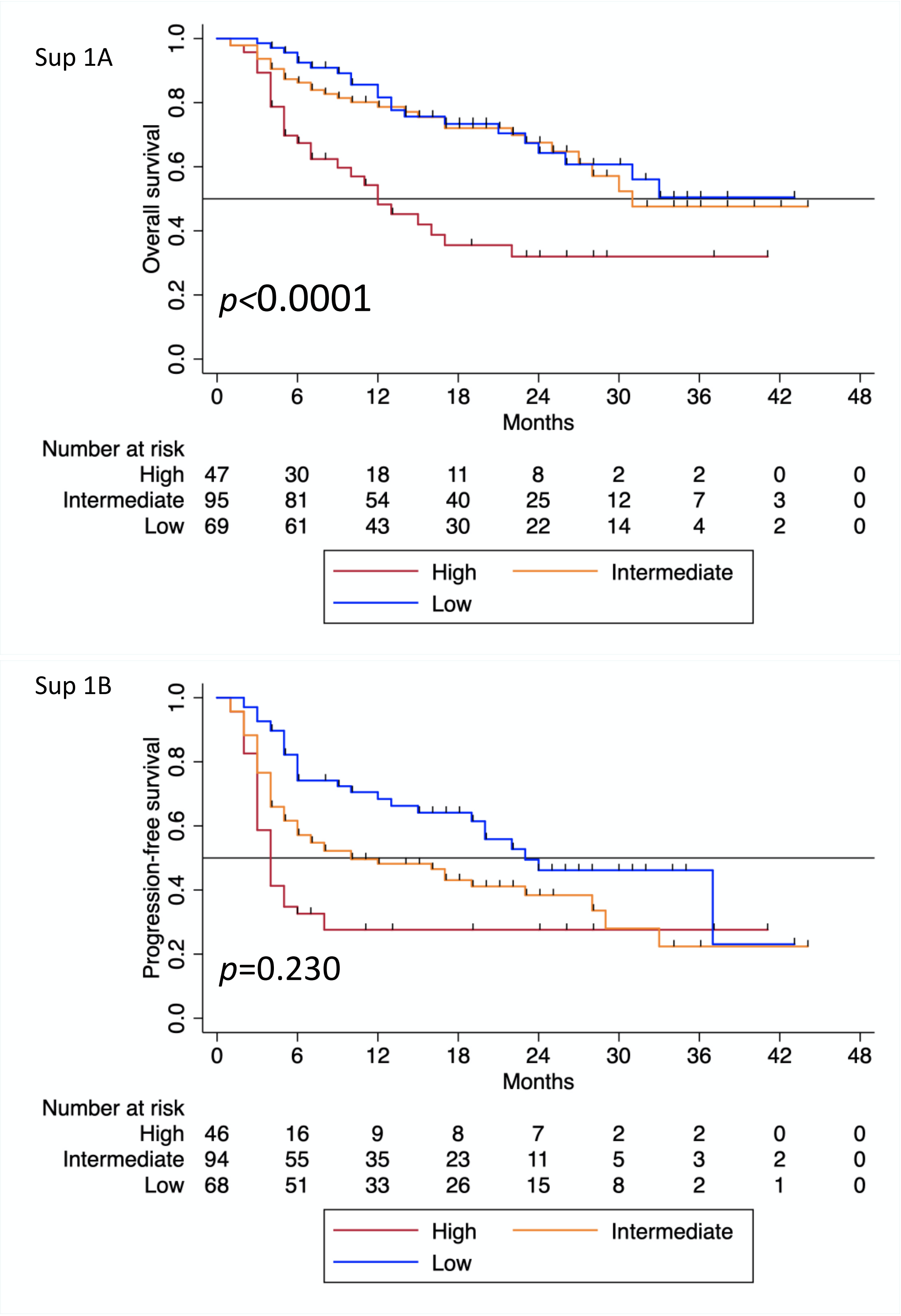

Figure 1. (**A**). Overall survival according to risk groups (low, intermediate and high) ($p < 0.0001$). The factors used were primary tumor localization and protein S-100B level. The subgroups were defined as follows: no risk factor (low), one risk factor (intermediate) and two risk factors (high). (**B**). Progression-free survival according to risk groups (low, intermediate and high) ($p = 0.230$). The factors used were primary tumor localization and protein S-100B level. The subgroups were defined as follows: no risk factor (low), one risk factor (intermediate) and two risk factors (high).

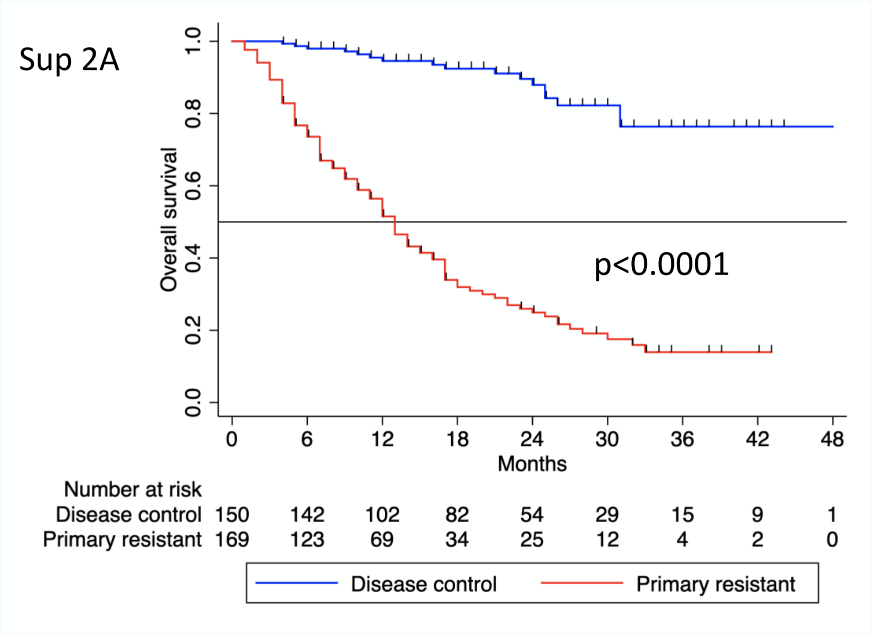

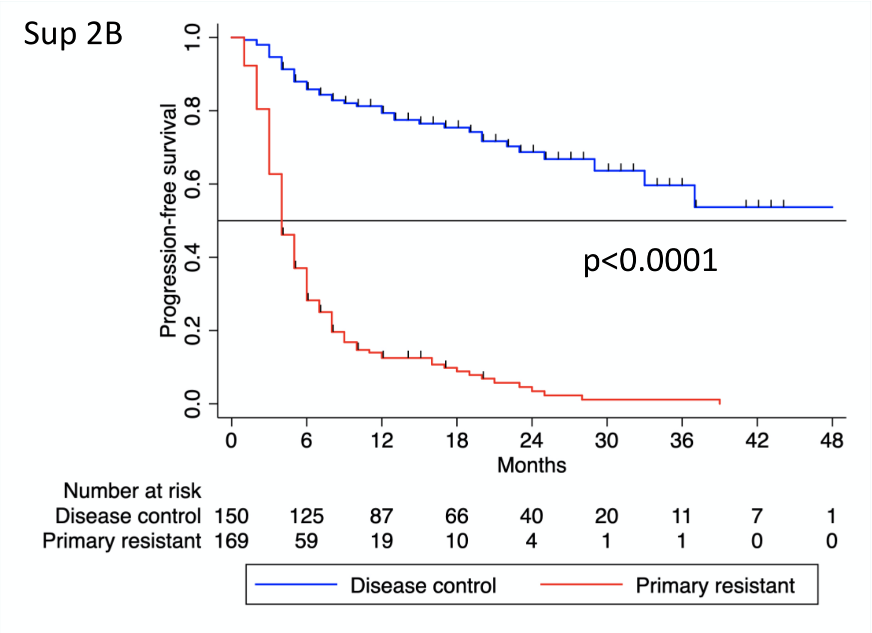

Figure S2. (**A**). Overall survival for the disease control group (complete response, partial response and stable disease for more than 6 months) and primary resistance (progressive disease and stable disease for less than 6 months). (**B**). Progression-free survival for the disease control group (complete response, partial response and stable disease for more than 6 months) and primary resistance (progressive disease and stable disease for less than 6 months).

Table S1. Patients characteristics and univariate analysis for the whole cohort. In this analysis, the primary resistance group includes progressive disease at the time of first tumor response evaluation after immunotherapy plus stable disease for less than 6 months. The disease control group includes complete response, partial response and stable disease for longer than 6 months.

Characteristics	ICI Cohort n = 319 N (%)	N (%) Primary Resistance PD, SD<6m n = 169 (47)	N (%) DC CR, PR, SD > 6 m n = 150 (63)	Univariate Analysis χ^2 test♣
**Age distribution**				
Median (years [IQR])	68 [56-77]	67 [54-78]	68 [57-77]	
<60y	101 (32)	54 (35)	47 (37)	0.947
60y-75y	114 (36)	59 (32)	55 (31)	
>75y	104 (32)	56 (33)	48 (32)	
**Sex**				
Male	192 (60)	93 (55)	99 (66)	**0.046**
Female	127 (40)	76 (45)	51 (34)	
**Tumor localization***				
Head and neck	54 (22)	18 (15)	36 (28)	
Trunk	73 (29)	32 (26)	41 (32)	**0.008**
Extremity	109 (43)	66 (53)	43 (34)	
Other	15 (6)	7 (6)	8 (6)	
**Histological subtype***				
SSM	76 (32)	42 (36)	34 (28)	
NM	72 (30)	27 (23)	45 (37)	
LMM	13 (6)	2 (2)	11 (9)	**0.004**
ALM	30 (12)	21 (17)	9 (8)	
Mucosal	15 (6)	7 (6)	8 (7)	
Other	32 (14)	19 (16)	13 (11)	
**Stage at initial diagnosis***				
I	48 (17)	26 (18)	22 (17)	
II	84 (31)	34 (24)	50 (38)	0.087
III	95 (35)	52 (37)	43 (32)	
IV	47 (17)	29 (21)	18 (14)	
**Number of organs with metastases**				
1-3	285 (89)	145 (86)	150 (93)	**0.030**
>3	34 (11)	24 (14)	10 (7)	
**Brain metastases**				
No	258 (81)	137 (81)	121 (81)	0.928
Yes	61 (19)	32 (19)	29 (19)	
**Liver metastases**				
No	204 (64)	102 (60)	102 (68)	0.156
Yes	115 (36)	67 (40)	48 (32)	
**BRAF mutation***				
BRAFmut	88 (45)	43 (43)	45 (47)	0.582
BRAFwt	106 (56)	56 (57)	50 (53)	
**LDH level***				
Normal	190 (68)	93 (64)	97 (72)	0.167
Elevated	90 (32)	52 (36)	38 (28)	
**S100B level***				
Normal	157 (56)	70 (48)	87 (64)	**0.010**
Elevated	125 (44)	75 (52)	50 (36)	

* patients with no information available were excluded in the respective analysis; m=months; IQR = interquartile range; ♣ Chi-square test performed between the two groups – primary resistance and disease control; ICI= immune-checkpoint inhibitors; y= years; SSM = superficial spreading melanoma; NM = nodular melanoma; LMM = lentigo malignant melanoma; ALM = acral lentiginous melanoma; BRAFmut = presence of BRAFV600E/K mutation; BRAFwt = BRAF wild-type; LDH = lactate dehydrogenase; S100B = tumor marker protein S100B.

Table S2. Patients characteristics and univariate analysis for the whole cohort according to type of first line immunotherapy.

Characteristics	ICI Cohort $n = 319$ n (%)	N (%) PD-1 Mono $n = 174$ (54.5)	PD-1 + CTLA4 $n = 145$ (45.5)	Univariate Analysis χ^2 test♣
**Age distribution**				
Median (years [IQR])	68 [56-77]	70 [59-79]	67 [52-75]	
<60y	104 (32)	44 (25.3)	57 (39.3)	**0.007**
60y-75y	117 (36)	62 (35.6)	52 (35.9)	
>75y	106 (32)	68 (39.1)	36 (24.8)	
**Sex**				
Male	195 (60)	109 (62.6)	83 (57.2)	0.326
Female	132 (40)	65 (37.4)	62 (42.8)	
**Tumor localization***				
Head and neck	54 (22)	32 (22.2)	22 (20.6)	
Trunk	73 (29)	41 (28.5)	32 (29.9)	0.864
Extremity	109 (43)	61 (42.4)	48 (44.9)	
Other	15 (6)	10 (6.9)	5 (4.7)	
**Histological subtype***				
SSM	76 (32)	36 (28.3)	40 (36)	
NM	72 (30)	45 (35.4)	27 (24.3)	
LMM	13 (6)	8 (6.3)	5 (4.5)	0.210
ALM	30 (12)	15 (11.8)	15 (13.5)	
Mucosal	15 (6)	10 (7.9)	5 (4.5)	
Other	32 (14)	13 (10.2)	19 (17.1)	
**Stage at initial diagnosis***				
I	48 (17)	28 (18.3)	20 (16.5)	
II	84 (31)	46 (30.1)	38 (31.4)	0.750
III	95 (35)	50 (32.7)	45 (37.2)	
IV	47 (17)	29 (19)	18 (14.9)	
**Number of organs with metastases**				
1-3	292 (89)	161 (92.5)	124 (85.5)	**0.043**
>3	35 (11)	13 (7.5)	21 (14.5)	
**Brain metastases**				
No	264 (81)	149 (85.6)	109 (75.2)	**0.018**
Yes	63 (19)	25 (14.4)	36 (24.8)	
**Liver metastases**				
No	209 (64)	118 (67.8)	86 (59.3)	0.115
Yes	118 (36)	56 (32.2)	59 (40.7)	
**BRAF mutation***				
BRAFmut	88 (45)	42 (36.5)	46 (58.2)	**0.003**
BRAFwt	106 (56)	73 (63.5)	33 (41.8)	
**LDH level***				
Normal	190 (68)	101 (70.1)	89 (65.4)	0.400
Elevated	90 (32)	43 (29.9)	90 (32.1)	
**S100B level***				
Normal	157 (56)	82 (55)	75 (56.4)	0.819
Elevated	125 (44)	67 (45)	58 (43.6)	

* patients with no information available were excluded in the respective analysis; IQR = interquartile range; ♣ χ^2 test performed between the two groups – PD-1 monotherapy and PD-1+CTLA4; ICI= immune-checkpoint inhibitors; y= years; SSM = superficial spreading melanoma; NM = nodular melanoma; LMM = lentigo malignant melanoma; ALM = acral lentiginous melanoma; BRAFmut = presence of BRAFV600E/K mutation; BRAFwt = BRAF wild-type; LDH = lactate dehydrogenase; S100B = tumor marker protein S100B

Table S3: Second line therapies in patients with primary resistance considering BRAF mutation status

BRAF Mutation Status	N (%)			
	IT (n = 25)	TT (n = 17)	CT (n = 21)	No Further Therapy (n = 64)
BRAFwt	10 (25)	0	10 (25)	20 (50)
BRAFV600E/K	3 (9.4)	17 (53.1)	1 (3.1)	11 (34.4)
Unknown	12 (21.8)	0	10 (18.2)	33 (60)

IT = immunotherapy (PD-1 monotherapy or PD-1+CTLA-4); TT = targeted therapy (BRAF inhibitors and MEK inhibitors) CT = chemotherapy

Table S4: Best overall response to second line therapies in patients with primary resistance considering BRAF mutation status

	N (%)			
	BRAFV600E/K Patients			
	CR	PR	SD	PD
IT n = 3	0	0	0	3 (100)
TT[a] n = 17	1 (6.3)	9 (56.3)	3 (18.7)	3 (18.7)
CT n = 1	0	0	0	1 (100)
	BRAFwt Patients			
IT n = 10[b]	0	0	3 (33)	6 (67)
CT n = 10[b]	0	1 (11)	0	8 (89)

IT = immunotherapy (PD-1 monotherapy or PD-1+CTLA-4); TT = targeted therapy (BRAF inhibitors and MEK inhibitors) CT = chemotherapy; [a] In 1 patient there was no information regarding the best overall response to the second line systemic therapy; [b] In 1 patient in the IT group and in the CT group, there was no information available on the best overall response to the second line systemic therapy
